# Septicæmia Hæmorrhagica

**Published:** 1896-07

**Authors:** 


SEPTICÆMIA HÆMORRHAGICA. D. J. Fisher (*De Veeartsenijkundige Bladen*, vol. ix. No. 3) describes under this name an epidemic which in the departments Buitenzorg and Meester Cornelis (in Dutch East India) carried off 658 buffaloes within seven months. It was asserted some time ago by Dr. van Éecke that the cattle-plague, peculiar to the so-called wild animals, and cattle epidemics often occurred in India; from the communication of Fisher it seems that this disease is indigenous in the districts mentioned.

It occurs in three forms: 1, the pectoral; 2, exanthematic; and, 3, the intestinal form. Often all three forms are present at the same time. The buffaloes suffered from tympanites (flatulent distention of the belly), with disturbed defecation and urination; in some cases diarrhœa was present. Respiration was frequent and painful. In other cases there was noticed extended swelling of the skin and subcuticle on the abdomen, breast, lower part of the neck, and a part of the legs; the eyeball was sunk in, the conjunctiva hyperæmic, the nose arid, the anus often open. The animals were always sleepy, refused all food, had high temperatures, and a painful and rapid respiration. In the pectoral and intestinal form at the post-mortem there were found a strong injection of the bloodvessels of the skin and minute hemorrhages in the subcuticle penetrating between the muscles. In the cavity of the chest there was much serofibrinous fluid, the whole surface of the pleura with the exception of the pleura phrenica was covered with a serofibrinous layer 3 cm. thick, which, after draining off the fluid, was only 1 mm. thick. The lungs were black-red, spotted or striped, not fallen together, soft, and easily torn, at which blood and the aforementioned fluid flowed off. The interstitial tissue, from 2 mm. to 1½ cm. in thickness, was strongly spotted, so that the lungs had a marble-like appearance. The tracheal and bronchial mucous membranes were likewise darkly colored or striped and covered with a foul layer of mucous. The pericardium, as a rule, contained little fluid, the wall of the heart was flaggy, and extended fibrinous coagulations were present in the heart.

The "rumen" (upper stomach of animals that chew the cud) was filled with dry food; here and there, through loss of epithelium, eroded and inflamed spots were found. The mucous membrane of the stomach (rennet) and intestines was dark-red, spotted, but without erosion. The Peyer glands were not especially swollen or areolated; the peritoneum was strongly injected; the liver was of normal size, but dark and soft; the gall-bladder was full and spotted red, the head of the spleen likewise, but otherwise normal; the kidneys plethoric and spotted black.

In the exanthematic form a fluid flowed out of the scarified and diseased parts of the skin corresponding to that in the cavity of the stomach; the subcuticle and the muscular tissue on the parts mentioned was saturated with this fluid matter, sometimes to the thickness of 10 cm. There were hemorrhages on the surfaces of the same. The whole was without odor. The lungs were in some cases unaffected, now and again hyperæmic; nose, mouth, and throat were considerably inflamed, as well as the mucous membrane of the genitalia. The intestinal changes were the same as those described above.

The contagion spread from from east to west, but not in the direction of the migration of the animals, which is from south to north. Fisher thinks that the wind had no influence on the spread of the disease, because great springs or leaps were often observed in its appearance. Just as little can the surface water be responsible, inasmuch as the disease arose at a dry season. The course of the rivers cannot serve as an explanation. The writer must leave the question unanswered how the disease arose and spread. The result of the inoculations made was not given. —W. C. S. in January *Tijdsch. voor Veeartsenijkunde*, etc.

---

A SERIES of articles are appearing in the current issues of the *Breeder's Gazette* on "Infectious Abortion in Cattle." They are written by a Mr. William Watson, a breeder. In the issue of May 13th the tenor of his whole claim as to the causation lies in the manure-piles and the drainings therefrom being taken into the affected animal's system.

A second writer on the same subject claims to have had very satisfactory results in feeding powdered gum-asafœtida in one to two tablespoonful doses, three times daily, where any evidence of aborting was present or where animals in previous years had aborted.

Germany demands more rigid inspection of American meat-products, because of its asserted admixture in transit with the meat-products of other countries. What next?

The second examination of the Pennsylvania State Board of Veterinary Medical Examiners proved a much more satisfactory one than the first one, and was very gratifying to the Board in that they were able to place their seal of approval on about 85 per cent. of the applicants.

Iodide of potash, in one-drachm doses three times daily, has almost completely reduced a schirrous cord with fistulous openings, from which a fetid discharge constantly emerged. Washing the parts locally with warm water lightly charged with creolin aided the rapid improvement at the Philadelphia Veterinary Sanitarium.

Dr. H. Walters, of Wilkesbarre, reports very satisfactory results with median neurotomy for ringbone. Out of over forty operated upon, but a small number have proven unsuccessful in removing entirely or greatly reducing the lameness.

